# Rapid Remission of Steroid-Refractory IgA Nephropathy With Targeted-Release Budesonide: A Case Report

**DOI:** 10.7759/cureus.107121

**Published:** 2026-04-15

**Authors:** Paola A Manrique-Pizarro, Emmanuel Cordero

**Affiliations:** 1 Internal Medicine, Universidad Autónoma de Guadalajara, Trujillo Alto, PRI; 2 Nephrology, Hospital Menonita de Guayama, Guayama, PRI

**Keywords:** chronic kidney disease (ckd), iga nephropathy (igan), massive proteinuria, steroid-refractory, targeted-release budesonide

## Abstract

IgA nephropathy (IgAN) is the most common primary glomerulonephritis worldwide and a major cause of progressive chronic kidney disease. Systemic corticosteroids are commonly used in high-risk patients but are frequently limited by inadequate response and significant adverse effects. We present the case of a 46-year-old woman with biopsy-confirmed IgAN who developed persistent microscopic hematuria and nephritic-range proteinuria. After six months of treatment with high-dose systemic prednisone, renin-angiotensin-aldosterone system blockade, and a sodium-glucose cotransporter-2 inhibitor, there was no clinical improvement, and she experienced marked cushingoid toxicity. Due to treatment failure and intolerance to systemic glucocorticoids, targeted-release budesonide was initiated. Over six months, proteinuria decreased from 2,536 mg/day to 66 mg/day, hematuria completely resolved, and estimated glomerular filtration rate improved from 65 to 80 mL/min/1.73 m². The treatment was well tolerated without recurrence of systemic steroid-related adverse effects. This case highlights targeted-release budesonide as an effective therapeutic option for selected patients with IgAN who fail or cannot tolerate systemic corticosteroid therapy.

## Introduction

IgA nephropathy (IgAN) is characterized by recurrent hematuria and variable proteinuria and is the most common primary glomerulonephritis worldwide [1,2]. Although some patients have a stable clinical course, up to 30-40% progress to chronic kidney disease or end-stage renal disease over time [1,2]. Diagnosis requires a renal biopsy, as the clinical presentation may overlap with other glomerular disorders.

Systemic corticosteroids are commonly prescribed in high-risk patients to reduce proteinuria and inflammation; however, limited efficacy and significant adverse effects remain major concerns [3-5]. Targeted-release budesonide is a gut-directed corticosteroid formulation designed to suppress mucosal production of galactose-deficient IgA1 (Gd-IgA1), a key driver of IgAN immunopathogenesis [1,2]. Randomized clinical trials have demonstrated clinically meaningful reductions in proteinuria and slowing of estimated glomerular filtration rate (eGFR) decline with this therapy [3-5].

We present a case of steroid-refractory IgAN with rapid and marked improvement following treatment with targeted-release budesonide.

## Case presentation

**Figure 1:**
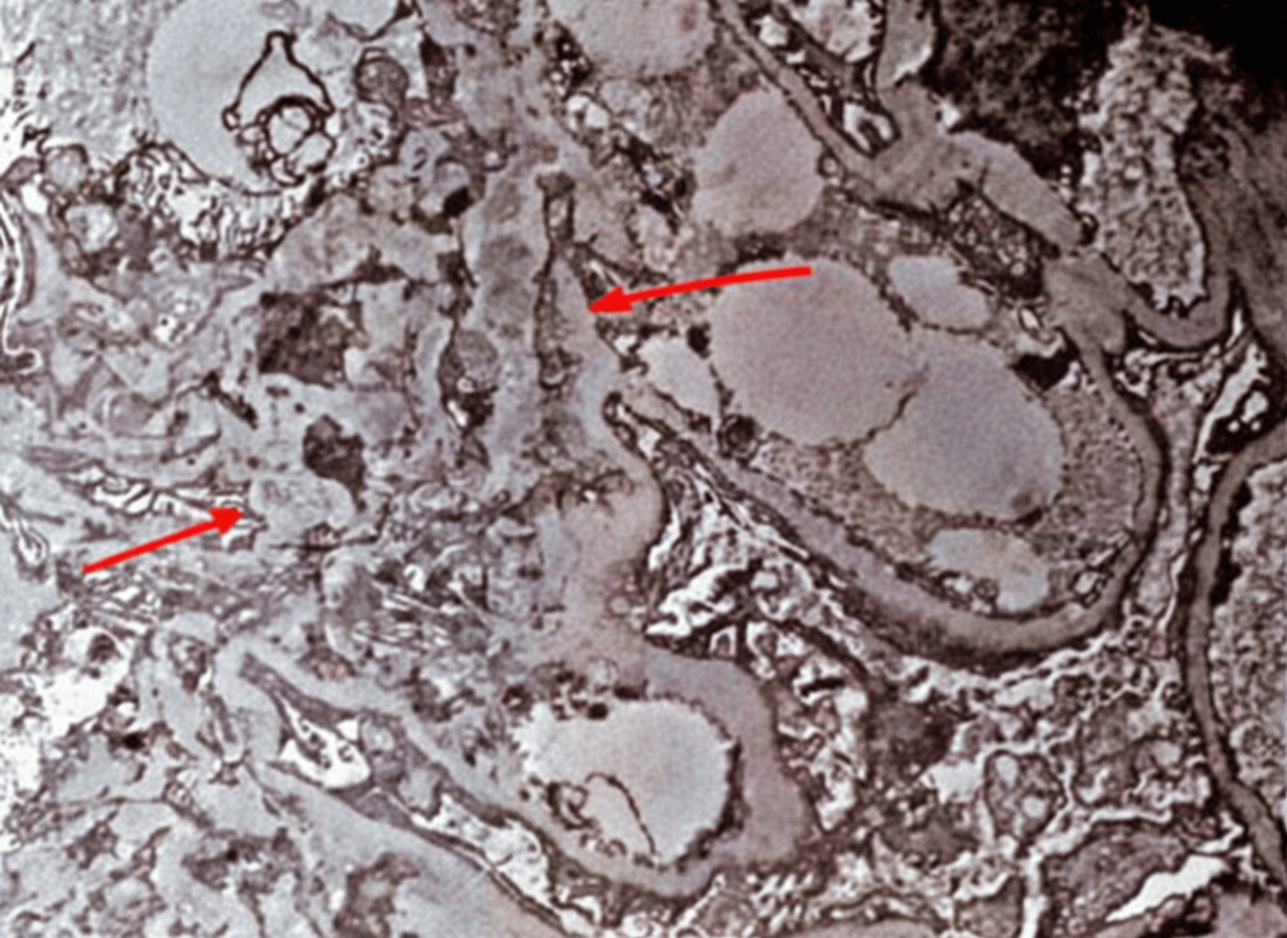
Electron microscopy of a kidney biopsy demonstrating mesangial immune-type electron-dense deposits (arrow), consistent with IgA nephropathy.

**Figure 2:**
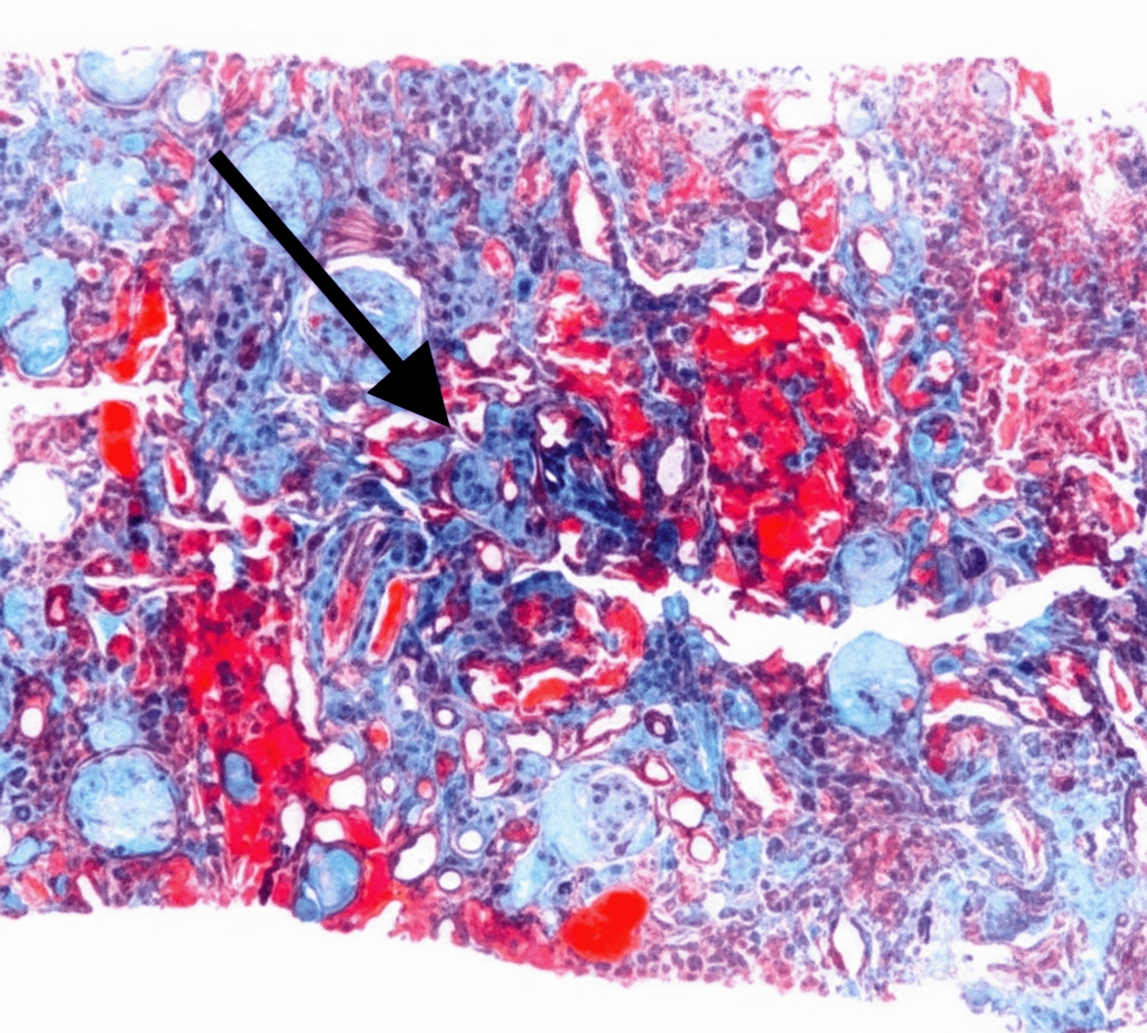
Renal biopsy demonstrating interstitial fibrosis and tubular atrophy consistent with chronic IgA nephropathy. Masson trichrome stain shows interstitial fibrosis (blue staining) with associated tubular atrophy.

A 46-year-old woman with a history of hypertension, gout, neuropathy, dyslipidemia, idiopathic intracranial hypertension status post-ventriculoperitoneal shunt placement, and epilepsy presented with hematuria and proteinuria while residing in Florida. Kidney biopsy demonstrated IgAN with segmental glomerulosclerosis and mild interstitial fibrosis and tubular atrophy, without mesangial or endocapillary hypercellularity or crescents. Electron microscopy revealed mesangial immune-type electron-dense deposits. Histopathologic findings are shown in Figure 1 and Figure 2.

The patient was treated with high-dose prednisone, losartan, and a sodium-glucose cotransporter 2 (SGLT2) inhibitor for six months. During therapy, she developed corticosteroid-related adverse effects, including Cushingoid facies and violaceous striae, without improvement in hematuria or proteinuria.

After relocating to Puerto Rico, nephrology evaluation revealed nephritic-range proteinuria of 2,536 mg/day, persistent microscopic hematuria, serum creatinine ranging from 1.07 to 1.23 mg/dL, and an eGFR of 55-65 mL/min/1.73 m². Given lack of clinical response and intolerance to systemic glucocorticoids, targeted-release budesonide was initiated in February 2025 at a dose of 16 mg orally once daily, administered as four 4 mg capsules taken in the morning at least one hour before meals, while renin-angiotensin-aldosterone system blockade and SGLT2 inhibitor therapy were continued.

Following initiation of targeted-release budesonide, renal parameters improved substantially. Serum creatinine decreased to 0.94 mg/dL, with a corresponding increase in eGFR to 76-81 mL/min/1.73 m². Proteinuria demonstrated a 97% reduction, with 24-hour urine protein decreasing from 2,536 mg/day to 66 mg/day, accompanied by complete resolution of microscopic hematuria. Overall, kidney function improved from chronic kidney disease stage 3a to stage 2. Table 1 summarizes renal function and urinary findings before and after budesonide therapy.

**Table 1:** Renal function and urinary findings before and after budesonide therapy. eGFR, estimated glomerular filtration rate; CKD, chronic kidney disease; UPCR, urine protein-to-creatinine ratio

Parameter	Pre-budesonide	Post-budesonide	Reference range	Clinical interpretation
Serum creatinine (mg/dL)	1.07-1.23	0.94	0.57-1.00	Improvement in renal function
eGFR (mL/min/1.73 m²)	55-65	76-81	≥59	CKD stage improved (G3a → G2)
Urine protein/creatinine ratio (mg/g)	2,536	424	<150	Marked proteinuria reduction
24-hour urine protein (mg/day)	2,536	66	<150	97% reduction
Urine albumin/creatinine ratio (mg/g)	174	198	<30	Persistent but reduced albuminuria
Microscopic hematuria	Present	Resolved	Absent	Resolution of glomerular inflammation

Given the lack of clinical response and intolerance to systemic glucocorticoids, targeted-release budesonide was initiated in February 2025 while renin-angiotensin-aldosterone system blockade and SGLT2 inhibitor therapy were continued. Over the following six months, hematuria resolved and proteinuria decreased by 97% (2,536 mg/day to 66 mg/day), achieving near-complete remission. eGFR improved to 80 mL/min/1.73 m² without recurrence of steroid-related adverse effects.

## Discussion

IgAN is driven by mesangial deposition of immune complexes containing Gd-IgA1, leading to complement activation, glomerular inflammation, and progressive renal injury [1,2]. Mucosal immune dysregulation plays a central role in this process, with Peyer’s patches in the distal ileum representing a major source of Gd-IgA1-producing plasma cells. Targeted-release budesonide is designed to deliver corticosteroid therapy directly to this gut-associated lymphoid tissue, thereby suppressing pathogenic IgA production while minimizing systemic corticosteroid exposure [1,3].

Despite optimized supportive care and prior systemic corticosteroid therapy, some patients with IgAN fail to achieve remission. This lack of response may be attributed to persistent mucosal immune activation and continued production of Gd-IgA1, which is not fully suppressed by systemic corticosteroids. Additionally, variability in individual responsiveness and the development of corticosteroid-related adverse effects may limit the effectiveness and tolerability of conventional therapy. These limitations highlight the need for targeted therapeutic strategies that directly address the underlying mucosal immune dysregulation.

Randomized trials, including NEFIGAN and NefIgArd, have demonstrated that targeted-release budesonide significantly reduces proteinuria and slows decline in eGFR in adults with IgAN at risk of progression [3-5]. These findings established the biological and clinical rationale for gut-directed immunomodulation in IgAN. Updated Kidney Disease: Improving Global Outcomes (KDIGO) guidelines now include targeted-release budesonide as a therapeutic option for patients with persistent proteinuria despite optimized supportive care [5].

The clinical course observed in our patient aligns with these trials, demonstrating a substantial reduction in proteinuria and improvement in kidney function following initiation of targeted-release budesonide. Notably, however, the magnitude of response in this case, near-complete remission of proteinuria with normalization of urinary sediment, exceeds the average reductions reported in randomized studies. This case, therefore, expands upon existing literature by illustrating that profound remission can occur even in steroid-refractory IgAN with established chronic histologic changes. Furthermore, successful treatment was achieved without recurrence of systemic glucocorticoid toxicity, highlighting the safety advantage of targeted mucosal therapy.

Overall, this report supports the growing evidence that targeted-release budesonide represents an effective and well-tolerated alternative for patients with IgAN who fail or cannot tolerate systemic glucocorticoids.

## Conclusions

Targeted-release budesonide may represent an effective therapeutic option for patients with IgAN who fail or cannot tolerate systemic corticosteroids. In this case, treatment was associated with improved proteinuria, resolution of hematuria, and improvement in kidney function despite prior steroid intolerance. These findings highlight the potential of gut-directed immunomodulation to achieve meaningful renal recovery while avoiding systemic glucocorticoid toxicity. Early biopsy confirmation, individualized risk assessment, and close monitoring of renal parameters remain essential to prevent irreversible renal decline and optimize long-term outcomes in IgAN.
